# Interplay of Semicoordination and π-Hole Bonding: The Case of Cocrystals of Group 10 (Ni, Pd, Pt) Dithiocarbonate Complexes with 1,4-Diiodotetrafluorobenzene

**DOI:** 10.3390/ijms27083668

**Published:** 2026-04-20

**Authors:** Marina A. Stozharova, Vitaly V. Suslonov, Rosa M. Gomila, Antonio Frontera, Anastasiya A. Eliseeva

**Affiliations:** 1 Institute of Chemistry, St. Petersburg State University, Universitetskaya Nab. 7/9, St. Petersburg 199034, Russia; 2 “TMH TM” LLC., Nepokoryonnykh Av. 47, St. Petersburg 195220, Russia; 3 Departament de Química, Universitat de les Illes Balears, Crta de Valldemossa km 7.5, 07122 Palma de Mallorca, Spain

**Keywords:** Group 10 metal, xanthate complexes, semicoordination, π-hole bonding, noncovalent interactions

## Abstract

A series of Group 10 metal dithiocarbonate complexes [M(S_2_CO*^i^*Pr)_2_] (M = Ni **1**, Pd **2**, Pt **3**) was prepared following procedures from the literature and cocrystallized with the ditopic σ/π-hole donor 1,4-diiodotetrafluorobenzene. Single-crystal X-ray diffraction revealed a consistent I···S halogen bonding motif alongside a remarkable diversity in metal-involving interactions across the Ni–Pd–Pt triad. While nickel(II) exhibits strong electrophilic M···S semicoordination, the palladium(II) center displays ambiphilic behavior, and platinum(II) acts exclusively as a nucleophile via π-hole···M bonding. Comprehensive density functional theory studies, including molecular electrostatic potential (MEP) mapping, quantum theory of atoms in molecules/noncovalent interaction plot analyses, and energy decomposition analysis, were used to quantify this competitive balance. The results demonstrate that the increasing nucleophilicity from Ni to Pt, supported by shifting MEP minima and stronger π-hole stabilization energies, dictates the preference for nucleophilic over electrophilic metal-centered contact.

## 1. Introduction

The concept of ambiphilicity—the ability to function as both an electron donor and acceptor—is well established in molecular chemistry [1,2,3] but has only recently been recognized for metal centers involved in noncovalent interactions [4,5]. Square planar d^8^ metal complexes exhibit electrophilic–nucleophilic dualism through their sterically accessible metal sites [6,7,8]. Their filled d_z_^2^-orbitals can serve as nucleophiles toward σ/π-hole donors, forming halogen bonds (HaBs) [9,10,11], chalcogen bonds [12,13,14], and pnictogen bonds [15,16], as well as engaging in π-hole···M interactions [17,18,19,20] with electron-deficient π-systems. Simultaneously, the vacant p_z_-orbitals of these d^8^ metals allow them to function as electrophiles, engaging in M∙∙∙X (X = S, Cl, I) semicoordination [21,22,23,24] with electron-rich atoms. This dual behavior implies that the type and directionality of metal-involving noncovalent interactions can be tuned.

Our investigation of the cocrystals of metal(II) dithiocarbamates [M(S_2_CNEt_2_)_2_] (M = Cu, Ni, Pd, Pt) with 1,3,5-triiodotrifluorobenzene revealed a progressive switch from M···I semicoordination (for Cu) to I···M HaBs (for Ni, Pd, and Pt) as the nucleophilicity of the metals increased across the series Cu–Ni–Pd–Pt [25]. A further study [26] on cocrystals of the complexes [M(S_2_CNEt_2_)_2_] (M = Ni, Pd, Pt) with 1,2-diiodoperfluorobenzene and 1,2-dibromoperfluorobenzene demonstrated that the {MS_4_} core also functions as an integrated π-hole acceptor toward electron-deficient π-systems of the arenes, engaging in π-hole···{MS_4_} interactions with essentially uniform behavior across the triad. A subsequent study [27] on the cocrystallization of [Pt(S_2_COEt)_2_] with fluorinated iodobenzenes showed that the geometry of the σ-hole donor (120° vs. 180°) can override the metal’s electronic preference, switching Pt^II^ between nucleophilic I···Pt HaB and electrophilic Pt···S semicoordination. Thus, while the transition from semicoordination to HaB across the Ni–Pd–Pt triad and the π-hole acceptor ability of the {MS_4_} core have been established, the interplay between electrophilic M···S semicoordination and nucleophilic π-hole···M bonding remains unexplored.

In this work, we address this question through a combined experimental and computational study of Group 10 metal dithiocarbonates [M(S_2_CO*^i^*Pr)_2_] (M = Ni **1**, Pd **2**, Pt **3**) cocrystallized with 1,4-diiodotetrafluorobenzene (1,4-FIB). The resulting cocrystals reveal a systematic progression of metal-involving noncovalent interactions across the triad: nickel(II) exhibits strong electrophilic M···S semicoordination, palladium(II) displays ambiphilic behavior, and platinum(II) acts exclusively as a nucleophile via π-hole···M bonding. A detailed computational study, including molecular electrostatic potential (MEP) surface analysis, quantum theory of atoms in molecules (QTAIM) and noncovalent interaction plot (NCIplot) analyses, and energy decomposition analysis (EDA), provides comprehensive insight into the physical origin and energetic features of the observed interactions. By integrating experimental and theoretical approaches, we establish a quantitative framework that explains how the electronic predisposition of the metal center governs the interplay between semicoordination and π-hole bonding across the Group 10 triad.

## 2. Results and Discussion

### 2.1. Cocrystal Growth and X-Ray Structures

**Figure 1:**
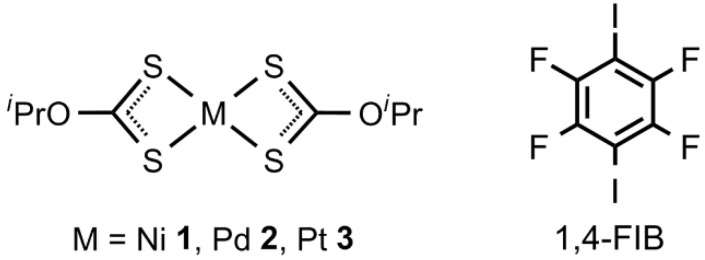
Dithiocarbonate complexes [M(S_2_CO*^i^*Pr)_2_] (M = Ni **1**, Pd **2**, Pt **3**) and the σ/π-hole donor 1,4-diiodotetrafluorobenzene used as coformers.

Cocrystallization of the dithiocarbonate complexes **1**–**3** and 1,4-FIB (Figure 1) from a 1:1 mixture in CH_2_Cl_2_/MeNO_2_ afforded crystalline adducts of **1**–**3** with 1,4-FIB. All these structures were established by single-crystal X-ray diffraction (XRD), with crystallographic data presented in Table 1 and Table S1.

Although the components were initially taken in equimolar amounts, the resulting cocrystals adopt distinct complex-to-donor stoichiometries, underscoring that the final cocrystal composition is dictated by the packing requirements and the distinct electronic characteristics of each metal center, which govern the competition between available intermolecular bonding motifs. For clarity, all three cocrystals are hereafter collectively denoted as (**1**–**3**)·1,4-FIB.

Cocrystal **1**·1,4-FIB crystallizes in the triclinic space group P-1. Its asymmetric unit comprises one 1,4-FIB molecule, one full molecule of **1**, and half of a second crystallographically independent complex molecule located on an inversion center, resulting in an overall 3:2 complex-to-donor ratio.

The palladium(II) analogue **2**·1,4-FIB is also triclinic (P-1) but contains one molecule of **2** per one half of 1,4-FIB located on an inversion center, resulting in a 2:1 stoichiometry. In contrast, **3**·1,4-FIB adopts the monoclinic space group C2/m, whose unit comprises one half of the platinum(II) complex and half of 1,4-FIB, both located on crystallographic mirror planes, affording a strict 1:1 complex-to-donor ratio.

### 2.2. Noncovalent Interactions in the Cocrystals

**Figure 2:**
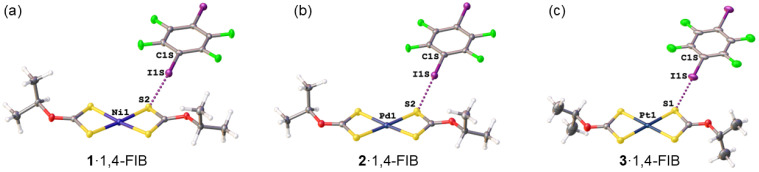
Ligand-centered I···S halogen bonds (HaBs) between iodine atoms of 1,4-diiodotetrafluorobenzene and sulfur atoms of the dithiocarbonate ligands in the structures of **1**·1,4-FIB (**a**), **2**·1,4-FIB (**b**), and **3**·1,4-FIB (**c**); HaBs are given by dotted lines, and thermal ellipsoids are shown with a 50% probability. Atom coloring: C grey, H white, O red, S yellow, F green, I purple, metal atoms (Ni, Pd, Pt) dark blue.

Despite these crystallographic differences, all three structures display strong I···S HaBs between the iodine atoms of 1,4-FIB and sulfur atoms of the dithiocarbonate ligands (Figure 2). The I···S distances range from 3.4052 to 3.4445 Å (Table 2), markedly shorter than the sum of Bondi van der Waals radii (Σ_vdW_; Σ_vdW_(I + S) = 3.78 Å) [28], while the C–I···S angles close to linear (175.85–179.35°), fulfilling the IUPAC criteria for HaBs [29,30]. This robust I···S motif serves as the primary supramolecular anchor in all three structures and mirrors the I···S HaBs observed in the previously reported cocrystals of ethyl nickel(II) [22] and platinum(II) [27] xanthates, as well as in cocrystals of the Group 10 metal dithiocarbamates [M(S_2_CNEt_2_)_2_] (M = Ni, Pd, Pt) with iodo- and bromo-substituted perfluoroarenes [25,26].

**Figure 3:**
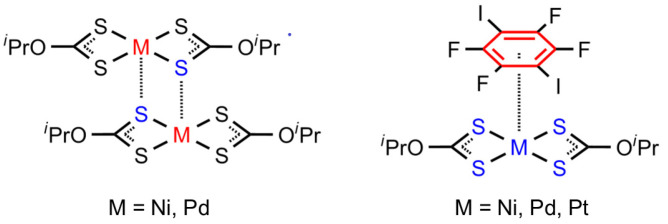
Schematic view of the metal-involving noncovalent interactions in the studied cocrystals: M···S semicoordination (**left**) and π-hole···{MS_4_} interactions (**right**).

While the I···S HaBs provide a consistent and robust structural framework across the cocrystals, the metal-involving noncovalent interactions exhibit a striking diversity. In **1**·1,4-FIB and **2**·1,4-FIB, the metal centers engage simultaneously in M···S semicoordination bonds (Figure 3, left) and π-hole···{MS_4_} interactions with 1,4-FIB (Figure 3, right). By contrast, in **3**·1,4-FIB, the platinum(II) center participates exclusively in π-hole···M interactions, with semicoordination completely suppressed.

The observed metal-involving interactions assemble the metal complexes and 1,4-FIB molecules into infinite parallel stacks through π-hole···{MS_4_} interactions (Figure S1). In **1**·1,4-FIB and **2**·1,4-FIB, adjacent complex molecules are further linked via mutual M···S contacts. This supramolecular motif is consolidated by several types of hydrogen bonds (HBs): C–H···S and C–H···O interactions interconnect the complexes, while C–H···F HBs link the complexes with 1,4-FIB (see Figure S2 and Table S3 in the Supporting Information). In the sections that follow, we examine each structure in detail, focusing on the geometric and energetic features of the metal-involving contacts.

#### 2.2.1. Metal-Involving Interactions in **1**·1,4-FIB

The crystal structure of **1**·1,4-FIB comprises two crystallographically independent molecules of **1**, hereafter referred to as Ni1 and Ni2. These two molecules display different modes of metal-involving noncovalent interactions.

**Figure 4:**
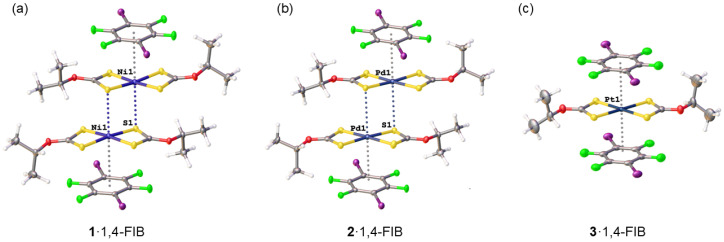
Metal-involving noncovalent interactions observed in the cocrystals: Ni···S semicoordination and π-hole···{NiS_4_} contacts in **1**·1,4-FIB (**a**), Pd···S semicoordination and π-hole···{PdS_4_} interactions in **2**·1,4-FIB (**b**), and π-hole···{PtS_4_} contacts in **3**·1,4-FIB (**c**). Atom coloring: C grey, H white, O red, S yellow, F green, I purple, metal atoms (Ni, Pd, Pt) dark blue. Short contacts are given by dotted lines. Thermal ellipsoids are shown with a 50% probability.

The Ni1 site forms a remarkably short Ni···S semicoordination bond with a sulfur atom of an adjacent complex molecule (Figure 4a). The Ni1···S1 distance (3.1706(5) Å) corresponds to a normalized contact (Nc) of 0.92, significantly below the Bondi Σ_vdW_ (Table 2). This separation is markedly shorter than the Ni···S contact observed in the ethyl analogue [Ni(S_2_COEt)_2_]·2(1,4-FIB) (3.496 Å) [22], indicating that the bulkier isopropyl substituents, when optimally oriented, do not hinder closer approach of the sulfur atom to the metal center.

Simultaneously, the Ni1 center participates in a weak π-hole···{MS_4_} interaction with the electron-deficient arene ring of 1,4-FIB. The Ni1···arene centroid distance (3.6367(7) Å) is greater than the corresponding Bondi Σ_vdW_ (3.33 Å) but remains within the crystallographic van der Waals sum proposed by Batsanov (3.70 Å) [31]. The C···S distances between 1,4-FIB and the {NiS_4_} core range from 3.6030(18) to 3.8283(19) Å, with a centroid-to-centroid NiS_4_/C_6_ separation of 3.5558(7) Å between the two planes (Table S2). The {NiS_4_} plane is tilted by a fold angle of 1.35(4)° and shifted laterally by 0.2878(10) Å relative to the arene plane, indicating a near-coplanar arrangement with only minor deviation. This offset reflects a collective π-hole···{MS_4_} interaction, where multiple S···C contacts accommodate the displacement, as supported by QTAIM analysis. Taken together, these geometric parameters are consistent with a π-hole···{MS_4_} interaction that, while clearly detectable, remains substantially weaker than the Ni···S semicoordination bond, as corroborated by theoretical considerations detailed in Section 2.3.

In contrast to Ni1, the second crystallographically independent Ni2 molecule, which is disordered, exhibits only weak π-hole···{MS_4_} interactions with 1,4-FIB (Figure S3, Table S4). Due to disorder of the isopropyl substituents in this molecule, detailed geometric parameters are provided in the Supporting Information (Section S4), and the computational analysis focuses on the Ni1 site, which demonstrates the key supramolecular behavior within the **1**·1,4-FIB cocrystal.

#### 2.2.2. Metal-Involving Interactions in **2**·1,4-FIB

The crystal structure of **2**·1,4-FIB contains one crystallographically independent palladium(II) complex molecule, which unites both electrophilic and nucleophilic bonding modes at a single metal center (Figure 4b). Similar to the Ni1 site, the palladium(II) center forms a semicoordination Pd···S contact with a sulfur atom of an adjacent complex molecule. The Pd1···S1 distance of 3.4495(10) Å is markedly longer (Nc = 1.01 vs. 0.92; Table 2) than the Ni···S separation observed in **1**·1,4-FIB, indicating a weaker electrophilic M···S interaction.

At the same time, the palladium(II) center participates in a π-hole···{PdS_4_} bonding with the electron-deficient π-system of 1,4-FIB, characterized by a Pd1···arene centroid distance of 3.6028(3) Å (Nc 1.08). This metal-involving interaction is accompanied by short C···S contacts between 1,4-FIB and the {PdS_4_} core, namely C2S···S1 (3.577(4) Å) and C3S···S3 (3.649(4) Å). The relative orientation of the {PdS_4_} and arene planes is characterized by a centroid-to-centroid distance of 3.5593(4) Å and a lateral shift of 0.2428(13) Å, which are nearly identical to those observed for Ni1 (Table S2). The lateral displacement indicates a collective π-hole···{PdS_4_} interaction, with QTAIM showing multiple S···C bond paths from the arene to several sulfur atoms but not directly to the metal center.

Thus, the geometric parameters of the observed metal-involving interactions in **2**·1,4-FIB demonstrate that the palladium(II) center exhibits a balanced ambiphilicity, acting simultaneously and with comparable strength as an electrophile (via Pd···S semicoordination) and as a nucleophile (via π-hole···{PdS_4_} bonding).

#### 2.2.3. Metal-Involving Interactions in **3**·1,4-FIB

In contrast to the ambiphilic palladium(II), the platinum(II) center in **3**·1,4-FIB is purely nucleophilic, engaging entirely in π-hole···{PtS_4_} interactions with 1,4-FIB (Figure 4c) and lacking any short Pt···S semicoordination contact. Instead, the molecules of **3** are separated by alternating 1,4-FIB molecules within perfectly aligned parallel stacks, confirming the complete suppression of the electrophilic bonding mode at the platinum(II) site (Figure S1).

The Pt1···arene centroid distance of 3.49505(15) Å is the shortest among the three cocrystals and approaches the Bondi Σ_vdW_ (3.5 Å), indicating an efficient π-hole···{PtS_4_} interaction. Remarkably, the {PtS_4_} and arene planes adopt a perfectly centered, parallel orientation with zero lateral shift (Table S2). This zero shift correlates with the direct C···Pt bond paths observed in QTAIM and with the strong nucleophilicity of Pt revealed by MEP (Section 2.3). The resulting ideal geometry maximizes contact between the electron-rich {PtS_4_} core and the π-hole, underscoring the particularly strong nucleophilic character of platinum(II) across the Ni–Pd–Pt triad.

These observations align with our previous findings for the platinum(II) dithiocarbamate complex [Pt(S_2_CNEt_2_)_2_] cocrystallized with 1,2-diiodotetrafluorobenzene [26], where the {PtS_4_} core participated in analogous π-hole bonding. This consistently high nucleophilicity of Pt in both ligand systems (dithiocarbamates vs. xanthates) is attributed to its more diffuse and energetically accessible d_z_^2^-orbital.

Thus, the exclusive nucleophilicity of the platinum(II) center in **3**·1,4-FIB contrasts with the ambiphilic behavior of the Ni1 and palladium(II) centers, reinforcing the progressive shift from electrophilic dominance to a pure nucleophilic character across the Group 10 triad. This experimentally observed trend provides a solid foundation for the theoretical analysis presented in Section 2.3, which quantifies the energetic and electronic factors governing the interplay between semicoordination and π-hole bonding.

### 2.3. Theoretical Considerations

To provide a deeper insight into the nature and energetic features of the observed noncovalent interactions, a comprehensive DFT study was performed. The analysis began with the calculation of MEP surfaces for 1,4-FIB and the complexes **1**–**3** to map the distribution of nucleophilic and electrophilic regions on the molecular surfaces. To characterize the stability and electronic properties of the specific supramolecular motifs, we conducted QTAIM and NCIplot analyses on the dimers featuring I···S HaBs, π-hole···{MS_4_} interactions, and M···S semicoordination. Furthermore, the physical origin of the primary structure-directing HaBs was investigated using EDA, while natural bond orbital (NBO) analysis was employed to quantify the charge transfer processes involved in these interactions. This theoretical framework allows for a quantitative comparison of the competitive balance between electrophilic and nucleophilic metal-centered contacts across the Ni–Pd–Pt triad.

**Figure 5:**
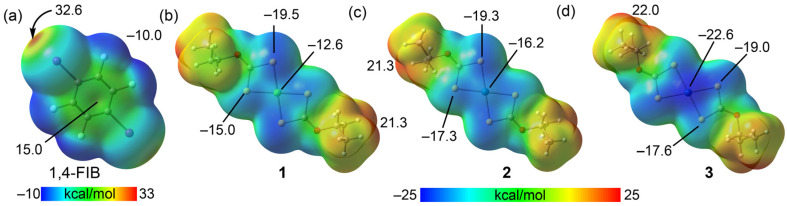
MEP surfaces of 1,4-diiodotetrafluorobenzene (**a**) and dithiocarbonate complexes [M(S_2_CO*^i^*Pr)_2_] where M is Ni (**b**), where M is Pd (**c**), and where M is Pt (**d**). The values of the MEP maxima and minima at the molecular surfaces are expressed in kcal/mol. Isosurface 0.001 a.u.

The MEP surface of 1,4-FIB reveals a prominent electrophilic region where the MEP maximum is located at the iodine atoms, exhibiting a deep σ-hole of 32.6 kcal/mol (Figure 5a). The aromatic ring also presents a positive potential of 15.0 kcal/mol, which corresponds to the π-hole. The MEP minimum is found at the fluorine atoms with a value of –10.0 kcal/mol.

The electrostatic surfaces of the Group 10 metal dithiocarbonate complexes **1**−**3** highlight the nucleophilic nature of the coordination core, with the MEP maxima located at the H-atoms of the isopropyl groups ranging from 21.3 to 22.0 kcal/mol (Figure 5b–d). In all three complexes, the entire {MS_4_} core is characterized by negative potential. For the Ni (**1**) and Pd (**2**) complexes, the MEP minima are located over the sulfur atoms (Figure 5b,c). Specifically, the S-atom with the longest M−S distance shows the most negative potential with values of −19.5 and −19.3 kcal/mol, respectively, while the S-atom with the shortest M−S distance is less nucleophilic at −15.0 and −17.0 kcal/mol, respectively. The nucleophilicity of the metal center increases moving down the group, with MEP values over the metal of −12.6 kcal/mol for Ni and −16.2 kcal/mol for Pd. In the platinum(II) complex (**3**), the MEP minimum shifts to the metal center itself at −22.6 kcal/mol, followed by the S-atom with the longest M−S distance at −19.0 kcal/mol and the S-atom with the shortest distance at −17.6 kcal/mol (Figure 5d). It is worth noting that the MEP at the S-atom with the longest M−S distance decreases upon going from Ni to Pt, while the MEP at the other S-atom becomes more negative, shifting from −15.0 to −17.6 kcal/mol, upon going from Ni to Pt, and the MEP at the metal center becomes significantly more negative, with the value increasing from −12.6 in Ni to −22.6 in Pt (Figure 5b–d).

**Figure 6:**
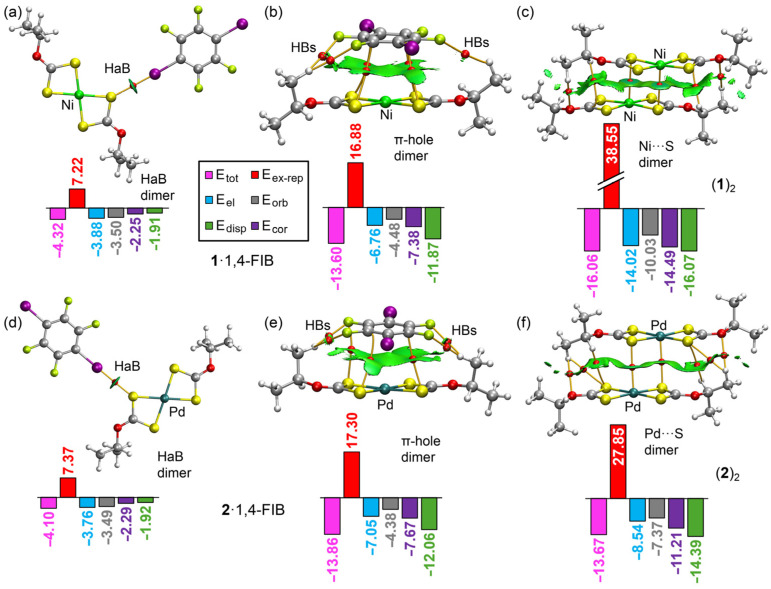
QTAIM and NCIplot analyses of representative dimers of the nickel(II) (top) and palladium(II) (bottom) complexes with 1,4-diiodotetrafluorobenzene or as homodimers. (**a**,**d**) halogen-bonded (HaB) dimers, (**b**,**e**) π-hole dimers, and (**c**,**f**) semicoordination M···S homodimers. Atom coloring: C grey, H white, O red, S yellow, F lime, I purple, Ni light green, Pd teal. Dimerization energies (E_tot_) and EDA terms are expressed in kcal/mol. Bond critical points are represented as red spheres and bond paths as orange lines.

The QTAIM and NCIplot analyses of the representative dimers for nickel(II) and palladium(II) are shown in Figure 6. The HaB dimers (Figure 6a,d) are characterized by a unique bond critical point (BCP, red sphere) and a bond path interconnecting the iodine atom of 1,4-FIB to the sulfur atom of the complex. This interaction is further visualized by a disk-shaped reduced density gradient (RDG) isosurface coincident with the location of the BCP. The dimerization energies are remarkably similar at −4.32 and −4.10 kcal/mol for the **1**·1,4-FIB and **2**·1,4-FIB complexes, respectively, which is consistent with the similar MEP values at the sulfur atoms in both complexes. The EDA indicates that the electrostatic (E_el_) and orbital (E_orb_) terms are the dominant attractive components in both complexes, followed by the correlation (E_corr_) and dispersion terms (E_disp_).

Regarding the π-hole dimers (Figure 6b,e), the QTAIM distribution reveals several BCPs and bond paths connecting 1,4-FIB to four sulfur atoms in the Ni complex and three sulfur atoms in the Pd complex. Additionally, several CH···F contacts further stabilize these assemblies, each characterized by a BCP, bond path and green RDG isosurface. Notably, no BCPs or bond paths were found connecting the metal centers directly to the 1,4-FIB molecule. However, the NCIplot displays an extended RDG isosurface between the aromatic ring and the {MS_4_} core, evidencing the π-hole···{MS_4_} nature of this interaction. The dimerization energies are nearly identical for both complexes, with values of −13.60 kcal/mol for Ni and −13.86 kcal/mol for palladium(II). EDA shows that dispersion and correlation terms are the primary attractive forces, in agreement with the π-nature of the interaction, while the orbital term is the least significant. The exchange-repulsion (E_ex-rep_) term is substantial in both dimers, around 17 kcal/mol, which significantly offsets the large dispersion and correlation contributions.

The QTAIM and NCIplot results for the semicoordination M···S homodimers are presented in Figure 6c,f. These short M···S contacts are characterized by BCPs and bond paths, along with bluish RDG isosurfaces. The dimers are further stabilized by an array of CH···C, CH···S, and CH···O contacts. In these homodimers, the Ni(II) complex exhibits a larger dimerization energy of −16.06 kcal/mol compared to −13.67 kcal/mol for palladium, which aligns with the shorter experimental distances observed for Ni. The EDA shows that all attractive terms are more pronounced for Ni, with dispersion and correlation being dominant. A key difference is the electrostatic term, which is considerably more negative for the Ni(II) dimer. The exceptionally large exchange repulsion observed in the nickel dimer, 38.55 kcal/mol, can be attributed to the short Ni···S experimental distance. For palladium(II), the stabilization energy of the homodimer is remarkably similar to that of the π-hole heterodimer. In contrast, for nickel(II), the homodimer is more energetically favorable, which is consistent with the lesser nucleophilicity of nickel(II) that favors semicoordination. This trend also explains the absence of a homodimer in the platinum(II) structure, as a hypothetical platinum(II) homodimer would likely be weaker than the palladium(II) analogue and therefore unable to compete with the π-hole heterodimer.

**Figure 7:**
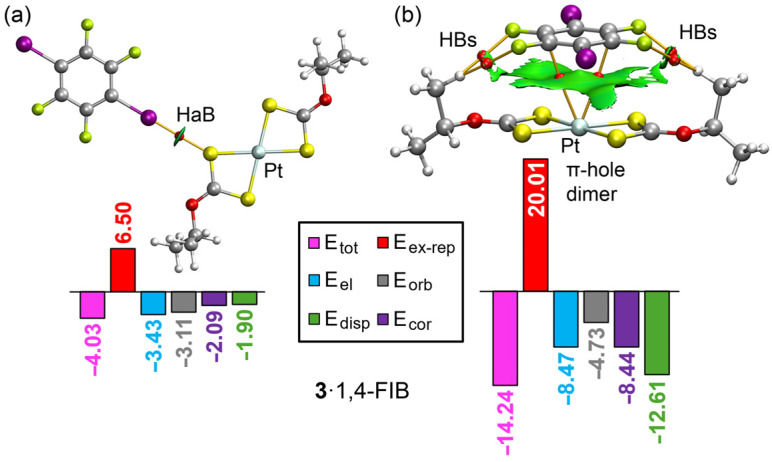
QTAIM and NCIplot analyses of representative dimers of the platinum(II) complex **3** with 1,4-FIB. Part (**a**) shows the halogen-bonded (HaB) dimer and part (**b**) shows the π-hole dimer. Atom coloring: C grey, H white, O red, S yellow, F lime, I purple, Pt silvery white. Dimerization energies (Etot) and EDA terms are expressed in kcal/mol. Bond critical points are represented as red spheres and bond paths as orange lines.

The QTAIM and NCIplot analyses for the **3**·1,4-FIB cocrystal are presented in Figure 7. The HaB dimer (Figure 7a) is very similar to those observed for **1**·1,4-FIB and **2**·1,4-FIB (Figure 6a,d), featuring an identical QTAIM and NCIplot distribution with a unique BCP and bond path between the iodine and sulfur atoms. The dimerization energy is slightly weaker at −4.03 kcal/mol, which is in good agreement with the MEP values at the sulfur atom of the platinum(II) complex (Figure 5d).

The most significant differences are found in the π-hole complex shown in Figure 7b. In this case, the Pt atom is directly connected by two BCPs and bond paths to two carbon atoms of the aromatic ring of 1,4-FIB. This finding strongly supports the MEP analysis, which showed that the potential minimum shifted from the sulfur atoms in complexes **1** and **2** to the metal center in complex **3**. The formation of this heterodimer is further assisted by four CH···F contacts, which are characterized by corresponding BCPs, bond paths, and green reduced density gradient isosurfaces. Notably, the dimerization energy for this π-hole assembly is −14.24 kcal/mol, which is larger than the values obtained for the **1**·1,4-FIB and **2**·1,4-FIB analogues. This increased stability explains the absence of homodimers in the **3**·1,4-FIB cocrystal. Furthermore, the electrostatic term in the platinum(II) π-hole dimer is more negative compared to the nickel(II) and palladium(II) dimers, aligning with the results of the MEP surface analysis.

**Figure 8:**
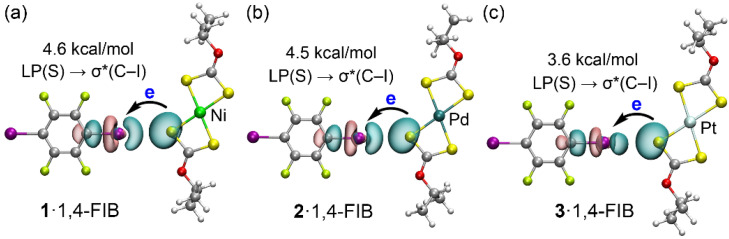
NBO analysis for the I···S halogen-bonded dimers of complexes **1**·1,4-FIB (**a**), **2**·1,4-FIB (**b**), and **3**·1,4-FIB (**c**) with 1,4-FIB. Atom coloring: C grey, H white, O red, S yellow, F lime, I purple, Ni light green, Pd teal, Pt silvery white. The donor–acceptor interactions and their corresponding second-order perturbation stabilization energies are indicated in kcal/mol.

NBO analysis was performed for the HaB dimers to investigate the orbital contributions to the supramolecular assembly. As shown in Figure 8, all three complexes exhibit a typical donor–acceptor interaction involving the lone pair (LP) of a sulfur atom, designated as LP(S), and the sigma antibonding orbital of the C–I bond, designated as σ*(C–I). This electron donation results in stabilization energies that range from 3.6 to 4.6 kcal/mol. Specifically, the strongest orbital stabilization is observed for the nickel(II) complex at 4.6 kcal/mol (Figure 8a), followed by the palladium(II) complex at 4.5 kcal/mol (Figure 8b), and the platinum(II) complex at 3.6 kcal/mol (Figure 8c). This trend is in excellent agreement with the molecular electrostatic potential analysis, the total interaction energies, and the orbital contributions calculated for the HaB dimers in Figure 6a,d and Figure 7a.

NBO analysis was also conducted for the π-hole dimers; however, no significant orbital contributions exceeding 0.5 kcal/mol were observed. Given that such values fall within the accuracy limits of the computational method, these results are not discussed further. This observation reinforces the energy decomposition analysis findings, which indicated that the π-hole interactions are primarily governed by dispersion and correlation forces rather than significant orbital overlap.

## 3. Materials and Methods

The complexes [M(S_2_CO*^i^*Pr)_2_] (M = Ni, Pd, Pt) were obtained following the general procedure [32]. Their full characterization has been reported previously [33,34,35]. Nickel(II) acetate tetrahydrate, potassium tetrachloropalladate(II), potassium tetrachloroplatinate(II), potassium isopropyl xanthate, 1,4-FIB, and all solvents were obtained from Sigma-Aldrich (Merck KGaA, Darmstadt, Germany) and used as received.

### 3.1. Cocrystal Growth

**1**·1,4-FIB. A solution of 1,4-FIB (12 mg, 0.03 mmol) in a chloroform/nitromethane mixture (1:1, *v*/*v*, 1 mL) was added to a solution of **1** (10 mg, 0.03 mmol) in the same mixture (1 mL), and then the formed solution was left to stand at room temperature (RT) for slow evaporation. Black plate-shaped crystals of **1**·1,4-FIB suitable for XRD were formed after 3–4 days.

**2**·1,4-FIB. A solution of 1,4-FIB (12 mg, 0.03 mmol) in a chloroform/nitromethane mixture (1:2, *v*/*v*, 1 mL) was added to a solution of **2** (11 mg, 0.03 mmol) in the same mixture (1 mL). Slow evaporation of the solvent at RT over 3–4 days afforded brownish-yellow crystals of **2**·1,4-FIB suitable for XRD.

**3**·1,4-FIB. A solution of 1,4-FIB (12 mg, 0.03 mmol) in a chloroform/nitromethane mixture (1:1, *v*/*v*, 1 mL) was added to a solution of **3** (14 mg, 0.03 mmol) in the same mixture (2 mL), and then the formed solution was left to stand at RT for slow evaporation. Yellow crystals of **3**·1,4-FIB suitable for XRD were released after 3–4 days.

### 3.2. X-Ray Structure Determinations

XRD data for single crystals of **1**·1,4-FIB and **2**·1,4-FIB were collected using an “XtaLAB Synergy” diffractometer (Rigaku Oxford diffraction, Wrocław, Poland) with Mo*Kα* (λ = 0.71073 Å) for **1**·1,4-FIB and Cu*Kα* radiation (λ = 1.54184 Å) for **2**·1,4-FIB. Suitable crystals of **3**·(1,4-FIB) were studied using a “SuperNova” diffractometer (Agilent Technologies, Santa Clara, CA, USA) with Mo*Kα* radiation (λ = 0.71073 Å). The crystals were kept at 100(2) K during all data collection. The structures were solved with the ShelXT structure solution program [36] using intrinsic phasing and refined by full-matrix least-squares on F^2^ with the ShelXL program [37], incorporated in the OLEX2 (v1.3) program package [38]. For **1**·1,4-FIB, disorder of the isopropyl groups (atoms C11–C14 in two orientations) in one of two crystallographically independent molecules of **1** was modeled over two alternative positions, with refined occupancies of 0.696 and 0.304. In **3**·1,4-FIB, the disordered isopropyl groups of **3** were modelled by splitting the affected atoms (C3 and C4) over two positions with equal site occupancy factors of 0.5:0.5. Hydrogen atoms were placed in calculated positions according to neutron diffraction statistical data and refined as riding atoms with isotropic displacement parameters [39].

Supplementary crystallographic data for this paper have been deposited at Cambridge Crystallographic Data Centre (CCDC 2538629, 2538631–2538632) and can be obtained free of charge via www.ccdc.cam.ac.uk/data_request/cif, accessed on 15 April 2026. Table with crystal data and structure refinement for 2538629, 2538631–2538632 can be found in Supporting Information.

### 3.3. Computational Details

The theoretical calculations were performed using the Turbomole 7.2 program package [40] at the PBE0-D4/def2-TZVP level of theory [41,42,43]. This density functional was combined with the D4 dispersion correction to accurately account for the long-range dispersion forces that are critical in noncovalent interactions [41]. For all complexes, the molecular electrostatic potential (MEP) surfaces were calculated and mapped onto the 0.001 a.u. electron density isosurfaces. To analyze the noncovalent contacts exactly as they exist in the solid state, all dimer calculations were performed using the coordinates obtained from the X-ray diffraction studies without further geometry optimization. The topological analysis of the electron density was carried out within the framework of the Quantum Theory of Atoms in Molecules (QTAIM) [44] and the Non-Covalent Interaction plot (NCIplot) [45] using the Multiwfn 3.8 software [46]. These methods allowed for the identification of bond critical points and the visualization of reduced density gradient (RDG) isosurfaces to characterize the nature of the supramolecular motifs. Natural Bond Orbital (NBO 7.0) [47] analysis was employed to quantify the second-order perturbation energies and charge transfer processes, specifically for the halogen-bonded dimers. Energy decomposition analysis (EDA) was performed [48,49] to partition the total dimerization energy into electrostatic, orbital, exchange-repulsion, dispersion, and correlation components. The visualization of molecular orbitals, QTAIM bond paths, and NCIplot isosurfaces was performed using the VMD 1.9.3 program [50].

## 4. Conclusions

This work presents the first systematic study of the interplay between electrophilic M···S semicoordination and nucleophilic π-hole···M bonding in the Group 10 triad, exemplified by a series of metal dithiocarbonate (M = Ni, Pd, Pt) complexes cocrystallized with 1,4-FIB. XRD analysis of the cocrystals revealed a clear and progressive shift in metal-centered bonding behavior: nickel(II) is dominated by strong electrophilic Ni···S semicoordination, palladium(II) displays balanced ambiphilicity, and platinum(II) acts exclusively as a nucleophile via π-hole···{PtS_4_} bonding, forming the shortest M···arene distance in the series.

MEP analysis confirmed there was increasing nucleophilicity down the group, with MEP values at the metal center becoming more negative from Ni^II^ (−12.6 kcal/mol) to Pt^II^ (−22.6 kcal/mol). The MEP minimum resides on sulfur atoms in the nickel(II) and palladium(II) complexes, favoring electrophilic M···S semicoordination, whereas for the platinum(II) analogue, it shifts to the metal center, thereby promoting nucleophilic π-hole···M interactions. For Ni^II^ and Pd^II^, π-hole bonding operates through the collective π-hole···{MS_4_} mechanism with multiple C···S bond paths; Pt^II^, in contrast, exhibits direct C···Pt contact, underscoring its enhanced nucleophilic character. The semicoordination homodimers destabilize progressively from Ni^II^ (−16.06 kcal/mol) to Pd^II^ (−13.67 kcal/mol), with Pt^II^ completely abandoning this bonding mode in favor of the more stable π-hole heterodimer (−14.24 kcal/mol). EDA revealed that HaBs are dominated by electrostatic and orbital contributions, while π-hole interactions are primarily stabilized by dispersion and correlation forces.

Thus, the experimental and computational results establish a quantitative framework demonstrating that the electronic predisposition of the metal center—specifically, the increasing nucleophilicity of the d_z_^2^-orbital and the shift of the MEP minimum from sulfur to the metal—governs the systematic transition from M···S semicoordination to π-hole···M bonding across the Group 10 triad. This explanation provides a predictive basis for tuning metal-involving interactions through metal selection, enabling the design of supramolecular systems with tailored electrophilic/nucleophilic behavior at the metal centers. The metal-dependent recognition of the σ/π-hole donor can be exploited for the development of chemical sensors, while the distinct reactivities of Ni (predominantly electrophilic) and Pt (predominantly nucleophilic) create opportunities for selective catalysis.

## Figures and Tables

**Table 1:** Crystal data for (**1**–**3**)·1,4-FIB.

	1·1,4-FIB	2·1,4-FIB	3·1,4-FIB
CCDC No.	2538629	2538631	2538632
Crystal system	triclinic	triclinic	monoclinic
Space group	P-1	P-1	C2/m
a/Å	9.65424(12)	9.3642(3)	17.9550(8)
b/Å	9.75898(13)	9.8595(3)	6.9901(3)
c/Å	16.7831(2)	10.7465(2)	9.5680(4)
*α*/°	106.8278(12)	104.294(2)	90
*β*/°	95.2642(10)	101.285(2)	103.156(4)
*γ*/°	103.0497(11)	103.829(3)	90
*V*/Å^3^	1453.35(3)	898.74(5)	1169.34(9)
*Z*	1	2	2

**Table 2:** Parameters of selected intermolecular contacts in the XRD structures of (**1**–**3**)·1,4-FIB.

Contact	Distance, Å (Nc) ^1^	Angle, °	Type of Contact
**1**·1,4-FIB			
C1S–I1S···S2 Ni1···S1	3.4383(5) (0.91) 3.1706(5) (0.92)	C1S–I1S–S2 176.19(5)	I···S HaB M···S semicoordination
π_(arene centroid)_···Ni1 C2S···S1 C3S···S2 C6S···S3 C5S···S4	3.6367(7) (1.09) 3.6030(18) (1.02) 3.8283(19) (1.09) 3.633(2) (1.04) 3.6609(18) (1.05)		π-hole···{MS_4_} interaction
**2**·1,4-FIB			
C1S–I1S···S2	3.4052(11) (0.90)	C1S–I1S–S2 175.85(11)	I···S HaB
Pd1···S1	3.4495(10) (1.01)		M···S semicoordination
π_(arene centroid)_···Pd1 C2S···S1 C3S···S2 C3S···S3	3.6028(3) (1.08) 3.577(4) (1.02) 3.875(4) (1.11) 3.649(4) (1.04)		π-hole···{MS_4_} interaction
C2S···S4	3.757(4) (1.07)		
**3**·1,4-FIB			
C1S–I1S···S1	3.4445(11) (0.91)	C1S–I1S–S1 179.35(16)	I···S HaB
π_(arene centroid)_···Pt1 C2S···S1	3.49505(15) (0.99) 3.577(4) (1.02)		π-hole···{MS_4_} interaction
C3S···S3	3.649(4) (1.04)		

^1^ Normalized contact (Nc) is defined as the ratio between the separation observed in the crystal and Bondi van der Waals sum ∑_vdW_ of interacting atoms: Nc = d/∑_vdW_; ∑_vdW_(I + S) = 3.78 Å, ∑_vdW_(Ni + S) = 3.43 Å, ∑_vdW_(Pd+ S) = 3.43 Å, ∑_vdW_(Ni + C) = 3.33 Å, ∑_vdW_(Pd + C) = 3.33 Å, ∑_vdW_(Pt + C) = 3.45 Å, ∑_vdW_(C + S) = 3.50 Å.

## Data Availability

The original contributions presented in this study are included in the article/Supplementary Material. Further inquiries can be directed to the corresponding author.

## Supplementary Materials

The following supporting information can be downloaded at https://www.mdpi.com/article/10.3390/ijms27083668/s1.

## Abbreviations

The following abbreviations are used in this manuscript:

HaB	Halogen bonding, halogen bond
1,4-FIB	1,4-diiodotetrafluorobenzene
MEP	Molecular electrostatic potential
QTAIM	Quantum theory of atoms in molecules
NCIplot	Noncovalent interaction plot
DFT	Density functional theory
EDA	Energy decomposition analysis
XRD	X-ray diffraction
∑_vdW_	Sum of van der Waals radii
HB	Hydrogen bond
NBO	Natural bond orbital
BCP	Bond critical point
RDG	Reduced density gradient
LP	Lone pair
RT	Room temperature
