# Supplementary material for: Interplay of Semicoordination and π-Hole Bonding: The Case of Cocrystals of Group 10 (Ni, Pd, Pt) Dithiocarbonate Complexes with 1,4-Diiodotetrafluorobenzene
Source: Int J Mol Sci. 2026 Apr 20;27(8):3668. doi: 10.3390/ijms27083668 (PMC13115864; doi:10.3390/ijms27083668)
Supplement: Supplementary file 1 [file ijms-27-03668-s001.zip › ijms-4239265-supplementary.pdf]

Electronic Supplementary Information

Interplay of Semicoordination and π-Hole Bonding: The Case of Cocrystals of Group 10 (Ni, Pd, Pt) Dithiocarbonate Complexes with 1,4-Diiodotetrafluorobenzene

Marina A. Stozharova [1], Vitaly V. Suslonov [1,2], Rosa M. Gomila [3], Antonio Frontera [3] and Anastasiya A. Eliseeva [1,*]

[1]*Institute of Chemistry, St. Petersburg State University, Universitetskaya Nab. 7/9, St. Petersburg 199034, Russia*

[2] *"TMH TM" LLC., Nepokoryonnykh Av. 47, St. Petersburg 195220, Russia*

[3]*Departament de Química, Universitat de les Illes Balears, Crta de Valldemossa km 7.5, 07122 Palma de Mallorca, SPAIN*

Table of Content

S1. Crystal data and strucrure refinement 3

S2. Crystal packing of (**1**–**3**)·1,4-FIB..4

S3. Hydrogen bonding in the cocrystals..5

S4. Noncovalent interactions involving Ni2 in **1**·1,4-FIB 6

S1. Crystal data and strucrure refinement

Table S1. Crystal data and structure refinement for (**1**–**3**)·1,4-FIB.

	**1**·1,4-FIB	**2**·1,4-FIB	**3**·1,4-FIB
CCDC No.	2538629	2538631	2538632
Empirical formula	$C_{36}H_{42}F_8I_4Ni_3O_6S_{12}$	$C_{11}H_{14}F_2IO_2PdS_4$	$C_{14}H_{14}F_4I_2O_2PtS_4$
M_W/g	1791.14	577.76	867.38
T/K	100(2)	100(2)	100(2)
Radiation	Mo*Kα* (λ = 0.71073)	Cu*Kα* (λ = 1.54184)	Mo *Kα* (λ = 0.71073)
Crystal color, shape	black, plate	brownish-yellow, prism	yellow, prism
Crystal size/mm^3	0.13 × 0.09 × 0.03	0.09 × 0.07 × 0.03	0.1 × 0.06 × 0.04
Crystal system	triclinic	triclinic	monoclinic
Space group	P-1	P-1	C2/m
a/Å	9.65424(12)	9.3642(3)	17.9550(8)
b/Å	9.75898(13)	9.8595(3)	6.9901(3)
c/Å	16.7831(2)	10.7465(2)	9.5680(4)
α/°	106.8278(12)	104.294(2)	90
β/°	95.2642(10)	101.285(2)	103.156(4)
γ/°	103.0497(11)	103.829(3)	90
V/Å^3	1453.35(3)	898.74(5)	1169.34(9)
Z	1	2	2
ρ_c/g·cm^{-3}	2.046	2.135	2.463
μ/mm^{-1}	3.582	26.344	9.036
F(000)	866.0	554.0	796.0
2Θ range/°	4.394 to 54.994	8.82 to 160.448	6.278 to 64.998
Reflections collected	34650	11644	3840
Independent reflections	6692 [R_{int} = 0.0278, R_{sigma} = 0.0215]	3731 [R_{int} = 0.0532, R_{sigma} = 0.0498]	2029 [R_{int} = 0.0293, R_{sigma} = 0.0354]
Data/restraints/parameters	6692/0/340	3731/0/194	2029/0/85
Goodness-of-fit on F^2	1.022	1.067	1.036
Final R indexes [I≥2σ (I)]	R_1 = 0.0189, wR_2 = 0.0421	R_1 = 0.0378, wR_2 = 0.1015	R_1 = 0.0264, wR_2 = 0.0593
Final R indexes [all data]	R_1 = 0.0231, wR_2 = 0.0435	R_1 = 0.0403, wR_2 = 0.1041	R_1 = 0.0283, wR_2 = 0.0604
Largest diff. peak/hole / e·Å^{-3}	0.49/-0.40	0.85/-1.62	1.49/-1.18

S2. Crystal packing of (1–3)·1,4-FIB

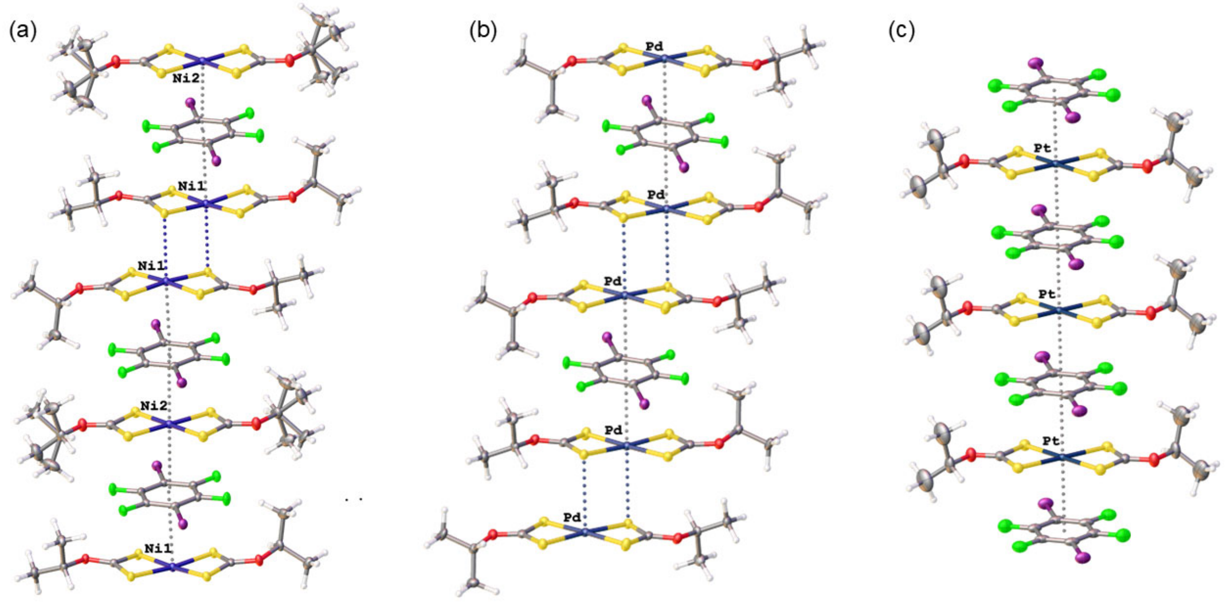

Figure S1. Fragment of crystal packing of **1**·1,4-FIB (a), **2**·1,4-FIB (b), and **3**·1,4-FIB (c), illustrating the alternating stacking motif of the metal complexes and 1,4-FIB. Short intermolecular interactions are given by dotted lines. Thermal ellipsoids are shown with a 50% probability. Atom coloring: C grey, H white, O red, S yellow, F green, I purple, metal atoms (Ni, Pd, Pt) dark blue.

Table S2. Plane-to-plane MS_4/C_6 distances, fold angles and lateral shifts in the XRD structures of (**1**–**3**)·1,4-FIB.

Cocrystal	Centroid-to-centroid distance, Å	Fold angle, °	Lateral shift, Å
**1**·1,4-FIB			
Ni1	3.5558(7)	1.35(4)	0.2878(10)
Ni2	3.4868(7)	0.37(5)	0.2488(11)
**2**·1,4-FIB	3.5593(4)	4.87(13)	0.2428(13)
**3**·1,4-FIB	3.49505(15)	0.00	0.0000

S3. Hydrogen bonding in the cocrystals

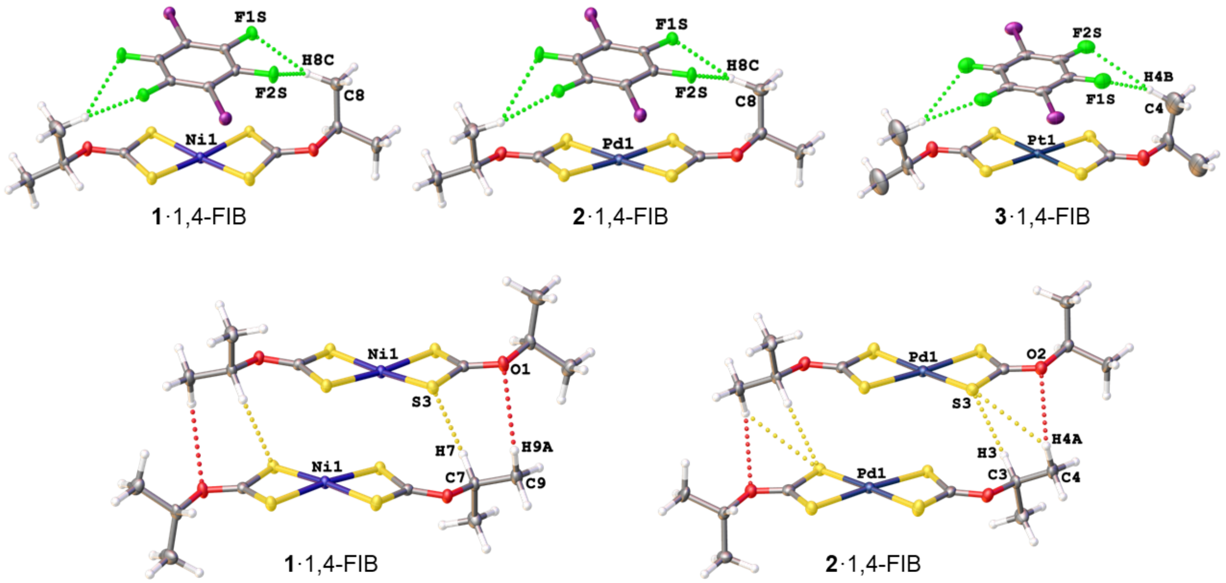

Figure S2. The C–H···F and C–H···X (X = S, O) hydrogen bonds (HBs) in hetero- (top) and homodimers (bottom) in the structures of (**1**–**3**)·1,4-FIB. The HBs are given by dotted lines. Thermal ellipsoids are shown with a 50% probability. Atom coloring: C grey, H white, O red, S yellow, F green, I purple, metal atoms (Ni, Pd, Pt) dark blue.

Table S3. Parameters of the C–H···X (X = F, S, O) HBs in the structures of (**1**–**3**)·1,4-FIB.

Structure	Contact	*d*(H···X), Å	*d*(C···X), Å	∠(C–H···X),°
1,4-FIB	C8–H8C···F1S	2.3996(12)	3.449(3)	160.06(12)
	C8–H8C···F2S	2.8383(13)	3.394(3)	111.36(13)
	C7–H7···S3	3.4530(5)	3.9201(19)	107.27(10)
	C9–H9A···O1	2.7637(14)	3.582(3)	131.34(12)
1,4-FIB	C8–H8C···F1S	2.457 (3)	3.518(6)	162.7(2)
	C8–H8C···F2S	3.008(3)	3.600(5)	114.3(3)
	C3–H3···S3	3.3846(9)	3.865(4)	107.9(2)
	C4–H4A···S3	3.173(1)	3.997(5)	132.6(2)
	C4–H4A···O2	2.657(3)	3.457(6)	129.4(3)
1,4-FIB	C4–H4B···F1S	2.810(3)	3.662(13)	134.7(6)
	C4–H4B···F2S	2.933(3)	4.015(14)	170.0(6)

S4. Noncovalent interactions involving Ni2 in 1·1,4-FIB

In **1**·1,4-FIB, the second independent complex molecule, Ni2, forms π-hole interactions with 1,4-FIB above and below the $\{NiS_4\}$ plane (**Figure S3b**). The Ni2···arene centroid distance of 3.4868(7) Å is shorter than that observed for Ni1 (3.6367(7) Å), but still longer than the corresponding sum of the Bondi van der Waals radii (Ni + C = 3.33 Å; **Table S3**). The $\{NiS_4\}$ core adopts a nearly parallel orientation with respect to the 1,4-FIB plane: the fold angle is close to zero (0.37(5)°), the centroid-to-centroid separation of 3.4868(7) Å, and the lateral shift diminishes to 0.2488(11) Å (**Table S3**). As in all three cocrystals, this π-hole interaction is accompanied by I···S halogen bonds between the iodine atoms of 1,4-FIB and sulfur atoms of the $\{NiS_4\}$ core (**Figure S3a**).

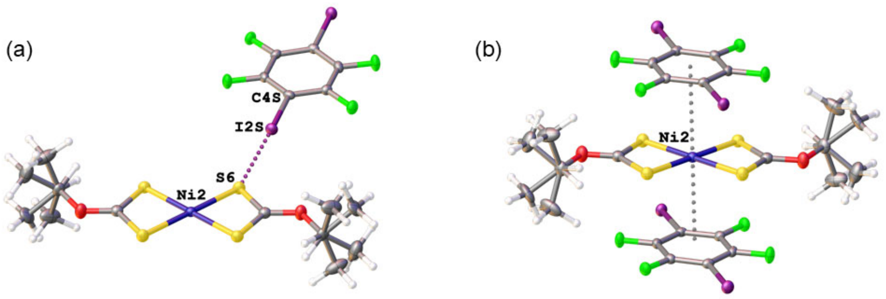

Figure S3. The I···S halogen bonds (a) and π-hole···$\{NiS_4\}$ interactions involving Ni2 molecule in the XRD structure of **1**·1,4-FIB. Short intermolecular contacts are given by dotted lines. Thermal ellipsoids are shown with a 50% probability. Atom coloring: C grey, H white, O red, S yellow, F green, I purple, Ni dark blue.

Table S4. Parameters of the I···S halogen bonds and π-hole···$\{MS_4\}$ interactions formed by Ni2 molecule in the crystal structure of **1**·1,4-FIB.

Contact	Distance, Å (Nc)[1]	Angle, °	Type of contact
**1**·1,4-FIB			
C4S–I2S···S6	3.4152(5) (0.90)	C4S–I2S–S6 176.24(5)	I···S HaB
$\pi_{(arene\ centroid)}$···Ni2	3.4868(7) (1.05)		π-hole···$\{MS_4\}$ interaction
C6S···S3	3.633(2) (1.04)		
C5S···S4	3.6609(18) (1.05)		

[1] Normalized contact (Nc) is defined as the ratio between the separation observed in the crystal and Bondi $\sum_{vdW}$ of interacting atoms: Nc = d/$\sum_{vdW}$; $\sum_{vdW}(I + S)$ = 3.78 Å, $\sum_{vdW}(Ni + C)$ = 3.33 Å, $\sum_{vdW}(C + S)$ = 3.50 Å
